# Gene expression analysis reveals the dysregulation of immune and metabolic pathways in Alzheimer's disease

**DOI:** 10.18632/oncotarget.12505

**Published:** 2016-10-06

**Authors:** Juan Chen, Chuncheng Xie, Yanhong Zhao, Zhiyan Li, Panpan Xu, Lifen Yao

**Affiliations:** ^1^ Department of Neurology, The First Affiliated Hospital, Harbin Medical University, Harbin, China; ^2^ Department of Neurosurgery, The First Affiliated Hospital, Harbin Medical University, Harbin, China; ^3^ Department of Internal Hematology, The First Affiliated Hospital, Harbin Medical University, Harbin, China

**Keywords:** Alzheimer's disease, pathway analysis, expression data, Pathology Section

## Abstract

In recent years, several pathway analyses of genome-wide association studies reported the involvement of metabolic and immune pathways in Alzheimer's disease (AD). Until now, the exact mechanisms of these pathways in AD are still unclear. Here, we conducted a pathway analysis of a whole genome AD case-control expression dataset (*n*=41, 25 AD cases and 16 controls) from the human temporal cortex tissue. Using the differently expressed AD genes, we identified significant KEGG pathways related to metabolism and immune processes. Using the up- and down- regulated AD gene list, we further found up-regulated AD gene were significantly enriched in immune and metabolic pathways. We further compare the immune and metabolic KEGG pathways from the expression dataset with those from previous GWAS datasets, and found that most of these pathways are shared in both GWAS and expression datasets.

## INTRODUCTION

To investigate Alzheimer's disease (AD) genetic risk factors, several genome-wide association studies (GWAS) have identified several new AD susceptibility loci in European populations, and some of these loci were successfully replicated in other studies [1-5]. To detect more AD genetic signals, several pathway analyses of GWAS datasets have been conducted [6-11]. Lambert et al. identified significant immune KEGG pathways and GO categories in AD [6]. Jones et al. reported significant KEGG pathways and 25 GO pathways in AD [8]. Most of the 25 pathways are related to metabolism [8]. Liu et al. highlighted the cardiovascular disease-related pathways in AD [12]. The International Genomics of Alzheimer's Project (IGAP) Consortium reported 10 significant KEGG pathways [13]. Until now, the potential mechanisms of these risk pathways in AD are still unclear.

We think that the different expression (up-regulation or down-regulation) of genes in these pathways may contribute to AD susceptibility. Here, we selected a whole genome AD case-control expression dataset (*n* = 41, 25 AD cases and 16 controls) from the human temporal cortex tissue, and conducted a pathway analysis using all the differently expressed AD genes, up-regulated AD genes, and down-regulated AD genes. We further compare the AD risk pathways from the expression dataset with those from previous GWAS datasets to address the potential mechanisms.

## RESULTS

### Pathway analysis of differently expressed AD genes

Using 1179 differently expressed AD genes, we identified 33 significant immune and metabolic pathways with *P* < 0.05 after FDR correction for multiple testing. These 33 pathways include 11 immune pathways and 22 metabolic pathways (Table 1). Oxidative phosphorylation (hsa00190) and Systemic lupus erythematosus (hsa05322) are the most significant metabolic and immune pathways with adjusted *P* = 1.33E-07 and *P* = 4.00E-04, respectively.

**Table 1:** significant pathways from the pathway analysis of differently expressed AD genes in expression dataset

Classification	Pathway ID	Pathway Name	C	O	E	R	rawP	adjP
Immunity	hsa05322	Systemic lupus erythematosus	136	13	3.72	3.5	9.70E-05	4.00E-04
Immunity	hsa05323	Rheumatoid arthritis	91	7	2.49	2.81	1.25E-02	1.70E-02
Immunity	hsa04666	Fc gamma R-mediated phagocytosis	94	9	2.57	3.5	1.10E-03	2.90E-03
Immunity	hsa04062	Chemokine signaling pathway	189	13	5.17	2.52	2.20E-03	4.80E-03
Immunity	hsa04640	Hematopoietic cell lineage	88	8	2.41	3.33	2.80E-03	5.50E-03
Immunity	hsa04650	Natural killer cell mediated cytotoxicity	136	10	3.72	2.69	4.30E-03	7.30E-03
Immunity	hsa04670	Leukocyte transendothelial migration	116	9	3.17	2.84	4.70E-03	7.80E-03
Immunity	hsa04660	T cell receptor signaling pathway	108	8	2.95	2.71	9.70E-03	1.41E-02
Immunity	hsa04622	RIG-I-like receptor signaling pathway	71	6	1.94	3.09	1.31E-02	1.75E-02
Immunity	hsa04662	B cell receptor signaling pathway	75	6	2.05	2.93	1.68E-02	2.22E-02
Immunity	hsa04612	Antigen processing and presentation	76	6	2.08	2.89	1.78E-02	2.29E-02
Metabolism	hsa00190	Oxidative phosphorylation	132	17	3.61	4.71	1.33E-07	2.19E-06
Metabolism	hsa00360	Phenylalanine metabolism	17	7	0.46	15.06	1.72E-07	2.43E-06
Metabolism	hsa00020	Citrate cycle (TCA cycle)	30	7	0.82	8.54	1.32E-05	9.48E-05
Metabolism	hsa00250	Alanine, aspartate and glutamate metabolism	32	7	0.87	8	2.07E-05	1.00E-04
Metabolism	hsa00830	Retinol metabolism	64	9	1.75	5.14	5.91E-05	3.00E-04
Metabolism	hsa00500	Starch and sucrose metabolism	54	8	1.48	5.42	1.00E-04	4.00E-04
Metabolism	hsa00350	Tyrosine metabolism	41	7	1.12	6.25	1.00E-04	4.00E-04
Metabolism	hsa00270	Cysteine and methionine metabolism	36	6	0.98	6.1	4.00E-04	1.30E-03
Metabolism	hsa00983	Drug metabolism - other enzymes	52	7	1.42	4.92	5.00E-04	1.50E-03
Metabolism	hsa00053	Ascorbate and aldarate metabolism	26	5	0.71	7.03	6.00E-04	1.70E-03
Metabolism	hsa00980	Metabolism of xenobiotics by cytochrome P450	71	8	1.94	4.12	7.00E-04	2.00E-03
Metabolism	hsa00982	Drug metabolism - cytochrome P450	73	8	2	4.01	9.00E-04	2.50E-03
Metabolism	hsa00040	Pentose and glucuronate interconversions	32	5	0.87	5.72	1.70E-03	3.90E-03
Metabolism	hsa00140	Steroid hormone biosynthesis	56	6	1.53	3.92	4.20E-03	7.30E-03
Metabolism	hsa00620	Pyruvate metabolism	40	5	1.09	4.57	4.50E-03	7.60E-03
Metabolism	hsa00380	Tryptophan metabolism	42	5	1.15	4.35	5.60E-03	8.90E-03
Metabolism	hsa00860	Porphyrin and chlorophyll metabolism	43	5	1.18	4.25	6.20E-03	9.60E-03
Metabolism	hsa00514	Other types of O-glycan biosynthesis	46	5	1.26	3.98	8.20E-03	1.23E-02
Metabolism	hsa00561	Glycerolipid metabolism	50	5	1.37	3.66	1.16E-02	1.59E-02
Metabolism	hsa00562	Inositol phosphate metabolism	57	5	1.56	3.21	1.96E-02	2.49E-02
Metabolism	hsa00010	Glycolysis / Gluconeogenesis	65	5	1.78	2.81	3.25E-02	3.83E-02
Metabolism	hsa00534	Glycosaminoglycan biosynthesis - heparan sulfate / heparin	26	5	0.71	7.03	6.00E-04	1.70E-03

C, the number of reference genes in the category; O, the number of genes in the gene set and also in the category; E, expected number in the category; R, the ratio of enrichment, rawP, the *p* value from hypergeometric test; adjP, the *p* value adjusted by the multiple test adjustment.

### Pathway analysis of up-regulated AD genes

Using 599 up-regulated AD genes, we identified 18 significant pathways including 6 immune and 12 metabolic pathways with *P* < 0.05 after FDR correction for multiple testing (Table 2). Systemic lupus erythematosus (hsa05322) and Phenylalanine metabolism (hsa00360) are the most significant immune and metabolic pathways with adjusted *P* = 1.44E-06 and *P* = 3.08E-05, respectively.

**Table 2:** significant pathways from the pathway analysis of up-regulated AD genes in expression dataset

Classification	Pathway ID	Pathway Name	C	O	E	R	rawP	adjP
Immunity	hsa05322	Systemic lupus erythematosus	136	13	1.89	6.88	6.41E-08	1.44E-06
Immunity	hsa04670	Leukocyte transendothelial migration	116	8	1.61	4.97	2.00E-04	7.00E-04
Immunity	hsa04640	Hematopoietic cell lineage	88	6	1.22	4.91	1.40E-03	2.40E-03
Immunity	hsa04650	Natural killer cell mediated cytotoxicity	136	7	1.89	3.71	3.00E-03	4.10E-03
Immunity	hsa04666	Fc gamma R-mediated phagocytosis	94	5	1.31	3.83	1.01E-02	1.23E-02
Immunity	hsa04660	T cell receptor signaling pathway	108	5	1.5	3.33	1.76E-02	2.03E-02
Metabolism	hsa00360	Phenylalanine metabolism	17	5	0.24	21.18	2.74E-06	3.08E-05
Metabolism	hsa00500	Starch and sucrose metabolism	54	7	0.75	9.33	9.69E-06	7.27E-05
Metabolism	hsa00053	Ascorbate and aldarate metabolism	26	5	0.36	13.85	2.63E-05	1.00E-04
Metabolism	hsa00830	Retinol metabolism	64	7	0.89	7.87	3.01E-05	2.00E-04
Metabolism	hsa00040	Pentose and glucuronate interconversions	32	5	0.44	11.25	7.51E-05	3.00E-04
Metabolism	hsa00860	Porphyrin and chlorophyll metabolism	43	5	0.6	8.37	3.00E-04	9.00E-04
Metabolism	hsa00350	Tyrosine metabolism	41	5	0.57	8.78	3.00E-04	9.00E-04
Metabolism	hsa00514	Other types of O-glycan biosynthesis	46	5	0.64	7.83	4.00E-04	1.00E-03
Metabolism	hsa00982	Drug metabolism - cytochrome P450	73	6	1.01	5.92	5.00E-04	1.10E-03
Metabolism	hsa00980	Metabolism of xenobiotics by cytochrome P450	71	6	0.99	6.08	5.00E-04	1.10E-03
Metabolism	hsa00983	Drug metabolism - other enzymes	52	5	0.72	6.92	8.00E-04	1.60E-03
Metabolism	hsa00140	Steroid hormone biosynthesis	56	5	0.78	6.43	1.10E-03	2.10E-03

C, O, E, R, rawP, and adjP have been defined in Table 1.

### Pathway analysis of down-regulated AD genes

Using 580 down-regulated AD genes, we identified 6 significant signals including 1 immune and 5 metabolic pathways with *P* < 0.05 after FDR correction for multiple testing (Table 3). Chemokine signaling pathway (hsa04062) is the only significant immune pathway with adjusted *P* = 1.90E-03. Interestingly, Oxidative phosphorylation (hsa00190), the most significant signal from the pathway analysis of all differently expressed AD genes (Table 1), is also the most significant metabolic pathway from pathway analysis of down-regulated AD genes with adjusted *P* = 2.99E-11 (Table 3).

**Table 3:** significant pathways from the pathway analysis of down-regulated AD genes in expression dataset

Classification	Pathway ID	Pathway Name	C	O	E	R	rawP	adjP
Immunity	hsa04062	Chemokine signaling pathway	189	9	2.54	3.54	1.10E-03	1.90E-03
Metabolism	hsa00190	Oxidative phosphorylation	132	17	1.78	9.58	3.15E-12	2.91E-11
Metabolism	hsa00020	Citrate cycle (TCA cycle)	30	7	0.4	17.35	1.19E-07	7.47E-07
Metabolism	hsa00250	Alanine, aspartate and glutamate metabolism	32	6	0.43	13.94	3.88E-06	1.62E-05
Metabolism	hsa00534	Glycosaminoglycan biosynthesis - heparan sulfate / heparin	26	5	0.35	14.3	2.25E-05	7.70E-05
Metabolism	hsa00230	Purine metabolism	162	7	2.18	3.21	6.60E-03	8.20E-05

C, O, E, R, rawP, and adjP have been defined in Table 1.

### Comparing the AD immune pathways using GWAS and expression data

We compared the 11 AD immune pathways with previous pathway analysis of AD GWAS. We found that 9 of the 11 pathways in Table 1 (excluding Leukocyte transendothelial migration, hsa04670 and B cell receptor signaling pathway, hsa04662) have been identified by Liu [12]. 4 of these 11 AD immune pathways including Natural killer cell mediated cytotoxicity (hsa04650), Antigen processing and presentation (hsa04622), RIG-I-like receptor signaling (hsa04612), and Hematopoietic cell lineage (hsa04640), have been reported [6, 8, 13].

### Comparing the AD metabolic pathways using GWAS and expression data

We compared the 22 AD metabolic pathways with previous pathway analysis of AD GWAS. We found that 10 of the 22 pathways in Table 1 have been identified [12], including Oxidative phosphorylation (hsa00190), Steroid hormone biosynthesis (hsa00140), Metabolism of xenobiotics by cytochrome P450 (hsa00980), Porphyrin and chlorophyll metabolism (hsa00860), Drug metabolism - cytochrome P450 (hsa00982), Pentose and glucuronate interconversions (hsa00040), Pyruvate metabolism (hsa00620), Retinol metabolism (hsa00830), Glycolysis / Gluconeogenesis (hsa00010), and Starch and sucrose metabolism (hsa00500).

## DISCUSSION

Recent studies reported the involvement of immune and metabolic KEGG pathways and GO categories in AD in European population [6, 8]. Evidence shows that the human genes may be differentially expressed in different ethnic populations [14]. Here, we investigated the potential mechanisms of these pathways in AD by a pathway analysis of a genome-wide expression dataset in European population [15]. In this pathway analysis, we analyzed all the differentially expressed genes, up-regulated genes and down-regulated genes [16]. It is reported that the analyzing the up- regulated genes and down-regulated genes is more powerful than analyzing all differentially expressed genes [16]. Using the differently expressed AD genes, we identified significantly enriched KEGG pathways related to metabolism and immune processes. Using the up-regulated and down-regulated AD gene list, we further found up-regulated AD genes were significantly enriched in immune and metabolic pathways. We further compare the immune and metabolic KEGG pathways from the expression dataset with those from previous GWAS datasets, and found that most of these pathways are shared in both GWAS and expression datasets.

Interestingly, the clinical studies supported the involvement of immune and metabolic processes in AD, such as disrupted energy metabolism [17-20], and dysregulation of iron metabolism [21], lipid metabolism [22], phosphoinositides and phosphatidic acid in AD [22], cerebellar glucose metabolism [23], hippocampal metabolism [24], and cerebral metabolism [25]. Take the Natural killer cell mediated cytotoxicity (hsa04650) for example. It is shared in the GWAS and expression datasets. Natural killer cells play an important role in host defence, and are involved in AD immunopathogenesis [26]. The increased natural killer cell cytotoxicity in AD may involve protein kinase C dysregulation [27]. Take the Oxidative phosphorylation (hsa00190) for example. Oxidative phosphorylation could influence clinical status and neuroimaging intermediates in AD [28], and involve early mitochondrial dysfunction and oxidative damage in AD [29]. In summary, our results show that the different expression of genes in immune and metabolic pathways may be associated with AD susceptibility. Our findings may offer new avenues for developing therapeutics for AD by regulating the expression of genes in these immune and metabolic pathways.

Meanwhile, some limitations exist in our research. Here, we performed the pathway analysis of AD expression data using the KEGG database and did not selected the GO categories, considering the difference between KEGG and GO databases [6, 8]. Here, we selected only 41 samples from the human temporal cortex tissue. In future, we will select more large-scale sample size and more brain regions to evaluate our findings, and further compare these findings with those from GO database.

## MATERIALS AND METHODS

### AD expression dataset

The expression dataset came from a whole genome AD case-control expression study (*n* = 41, 25 cases and 16 controls) using human temporal cortex tissue [15]. 25 AD cases and 16 controls were well-matched for age and postmortem delay [15]. We got 1196 significantly differently expressed Affymetrix transcripts with *P* < 0.05 after false discovery rate (FDR) correction [15].

### Dataset preprocessing

In this expression dataset, the 1196 significantly differently expressed transcripts correspond to 1361 unique genes. 1179 of 1361 genes were successfully mapped to 1179 unique Entrez Gene IDs in WebGestalt database [30]. Other 182 genes were mapped to multiple Entrez Gene IDs or could not be mapped to any Entrez Gene ID. The following pathway analysis of all the differently expressed genes will be based upon the 1179 unique genes.

The total of 1196 significantly differently expressed transcripts include 603 significantly up-regulated (AD cases *vs*. controls) and 593 significantly down-regulated (AD cases *vs*. controls) transcripts, which correspond to 710 and 654 unique genes (3 transcripts are both up- and down-regulated in AD cases and controls). 599 of 710 up-regulated genes were successfully mapped to 599 unique Entrez Gene IDs in WebGestalt database [30]. Other 111 genes were mapped to multiple Entrez Gene IDs or could not be mapped to any Entrez Gene ID. The following pathway analysis of up-regulated genes will be based upon the 599 unique genes. 580 of 654 up-regulated genes were successfully mapped to 580 unique Entrez Gene IDs in WebGestalt database [30]. Other 74 genes were mapped to multiple Entrez Gene IDs or could not be mapped to any Entrez Gene ID. The following pathway analysis of down-regulated genes will be based upon the 580 unique genes.

### Pathway-based test

We selected the WebGestalt database for pathway analysis, and the hypergeometric test was used to identify the significant KEGG pathways [30]. We first conducted a pathway analysis using all the differently expressed AD genes, significantly up-regulated (AD cases *vs*. controls) and down-regulated genes (AD cases *vs*. controls). We selected KEGG pathways including 10-300 genes for subsequent pathway analysis [31]. The entire Entrez gene set was defined to be the reference gene list [30]. We limited potential KEGG pathways with least AD risk 5 genes and with FDR adjusted *p*-value below 0.05 [30].

## Supplementary Material


