# Supplementary material for: Gene expression analysis reveals the dysregulation of immune and metabolic pathways in Alzheimer's disease
Source: Oncotarget. 2016 Oct 6;7(45):72469–74. doi: 10.18632/oncotarget.12505 (PMC5341922; doi:10.18632/oncotarget.12505)
Supplement: Supplementary file 1 [file oncotarget-07-72469-s001.pdf]

Gene expression analysis reveals the dysregulation of immune and metabolic pathways in Alzheimer's disease

Supplementary Material

Supplementary Table 1, Gene IDs in pathway analysis of all differently expressed AD genes in expression dataset

Classifications	Pathway Name	Pathway ID	Entrez ID
Immune diseases	Systemic lupus erythematosus	hsa05322	8968 8352 733 8341 8351 8350 8358 8353 8355 8357 8369 8356 8354
Immune diseases	Rheumatoid arthritis	hsa05323	1514 7040 529 1435 284 534 9550
Immune system	Fc gamma R-mediated phagocytosis	hsa04666	5581 2934 5335 4651 8976 3984 5580 7410 8394
Immune system	Chemokine signaling pathway	hsa04062	5829 8976 5580 7410 5590 2185 2932 409 23236 5566 5970 56288 5567
Immune system	Hematopoietic cell lineage	hsa04640	3554 4254 960 909 1435 7037 914 945
Immune system	Natural killer cell mediated cytotoxicity	hsa04650	5535 3824 5335 4068 7410 5534 2185 5533 8797 3107
Immune system	Leukocyte transendothelial migration	hsa04670	2185 5335 5829 1365 9080 399 83700 7410 49861
Immune system	T cell receptor signaling pathway	hsa04660	5535 5335 7410 868 5534 2932 5533 5970
Immune system	RIG-I-like receptor signaling pathway	hsa04622	3661 9474 5970 5300 7186 8717
Immune system	B cell receptor signaling pathway	hsa04662	5535 2932 5533 5970 7410 5534
Immune system	Antigen processing and presentation	hsa04612	1514 3824 5993 3107 5641 4802
Metabolism	Oxidative phosphorylation	hsa00190	4704 4719 7384 506 4701 6389 509 534 27068 7385 4722 4715 498 529 4702 4720 9550
Metabolism	Phenylalanine metabolism	hsa00360	259307 6898 2805 2806 314 1644 218
Metabolism	Citrate cycle (TCA cycle)	hsa00020	5160 4190 1738 6389 55753 47 1737
Metabolism	Alanine, aspartate and glutamate metabolism	hsa00250	259307 2572 2805 56954 2571 2806 27165
Metabolism	Retinol metabolism	hsa00830	56603 10720 54490 157506 53630 79799 7364 51109 7365
Metabolism	Starch and sucrose metabolism	hsa00500	8972 79799 178 10720 7364 54490 7365 6476
Metabolism	Tyrosine metabolism	hsa00350	259307 6898 2805 2806 314 1644 218
Metabolism	Cysteine and methionine metabolism	hsa00270	259307 6898 27430 2805 2806 4357
Metabolism	Drug metabolism - other enzymes	hsa00983	8833 3251 79799 10720 7364 54490 7365
Metabolism	Ascorbate and aldarate metabolism	hsa00053	79799 10720 7364 54490 7365
Metabolism	Metabolism of xenobiotics by cytochrome P450	hsa00980	10720 54490 218 79799 2941 9446 7364 7365
Metabolism	Drug metabolism - cytochrome P450	hsa00982	10720 54490 218 79799 2941 9446 7364 7365
Metabolism	Pentose and glucuronate interconversions	hsa00040	79799 10720 7364 54490 7365
Metabolism	Steroid hormone biosynthesis	hsa00140	79799 412 10720 7364 54490 7365
Metabolism	Pyruvate metabolism	hsa00620	5160 4190 32 1738 1737
Metabolism	Tryptophan metabolism	hsa00380	259307 56267 1644 55753 15

Metabolism	Porphyrin and chlorophyll metabolism	hsa00860	79799 10720 7364 54490 7365
Metabolism	Other types of O-glycan biosynthesis	hsa00514	79799 10720 7364 54490 7365
Metabolism	Glycerolipid metabolism	hsa00561	160851 4023 55750 1608 9162
Metabolism	Inositol phosphate metabolism	hsa00562	3707 5335 8394 23236 51196
Metabolism	Glycolysis / Gluconeogenesis	hsa00010	5160 1738 5213 218 1737
Metabolism	Glycosaminoglycan biosynthesis - heparan sulfate / heparin	hsa00534	90161 9348 2132 2135 26035

Supplementary Table 2, Gene IDs in pathway analysis of up-regulated AD genes in expression dataset

Classifications	Pathway Name	Pathway ID	Entrez ID
Immune diseases	Systemic lupus erythematosus	hsa05322	733 8968 8352 8341 8351 8358 8350 8353 8355 8357 8369 8356 8354
Immune system	Leukocyte transendothelial migration	hsa04670	5335 5829 9080 7410 49861 1365 399 83700
Immune system	Hematopoietic cell lineage	hsa04640	3554 960 909 1435 914 945
Immune system	Natural killer cell mediated cytotoxicity	hsa04650	5535 3824 5335 8797 3107 4068 7410
Immune system	Fc gamma R-mediated phagocytosis	hsa04666	2934 5335 8394 4651 7410
Immune system	T cell receptor signaling pathway	hsa04660	5535 5335 5970 7410 868
Metabolism	Phenylalanine metabolism	hsa00360	259307 6898 314 1644 218
Metabolism	Starch and sucrose metabolism	hsa00500	8972 79799 7364 10720 54490 7365 6476
Metabolism	Ascorbate and aldarate metabolism	hsa00053	79799 7364 10720 54490 7365
Metabolism	Retinol metabolism	hsa00830	53630 79799 7364 10720 54490 157506 7365
Metabolism	Pentose and glucuronate interconversions	hsa00040	79799 7364 10720 54490 7365
Metabolism	Porphyrin and chlorophyll metabolism	hsa00860	79799 7364 10720 54490 7365
Metabolism	Tyrosine metabolism	hsa00350	259307 6898 314 1644 218
Metabolism	Other types of O-glycan biosynthesis	hsa00514	79799 7364 10720 54490 7365
Metabolism	Drug metabolism - cytochrome P450	hsa00982	79799 7364 10720 54490 218 7365
Metabolism	Metabolism of xenobiotics by cytochrome P450	hsa00980	79799 7364 10720 54490 218 7365
Metabolism	Drug metabolism - other enzymes	hsa00983	79799 7364 10720 54490 7365
Metabolism	Steroid hormone biosynthesis	hsa00140	79799 7364 10720 54490 7365

Supplementary Table 3, Gene IDs in pathway analysis of down-regulated AD genes in expression dataset

Classifications	Pathway Name	Pathway ID	Entrez ID
Immune system	Chemokine signaling pathway	hsa04062	8976 5580 5590 2185 2932 409 23236 5566 5567 4704 4719 7384 506 4701 6389 534 509 7385 27068 4722 4715
Metabolism	Oxidative phosphorylation	hsa00190	498 4702 529 4720 9550
Metabolism	Citrate cycle (TCA cycle)	hsa00020	5160 4190 1738 6389 55753 47 1737
Metabolism	Alanine, aspartate and glutamate metabolism	hsa00250	2572 2805 56954 27165 2571 2806
Metabolism	Glycosaminoglycan biosynthesis - heparan sulfate / heparin	hsa00534	90161 9348 2132 2135 26035
Metabolism	Purine metabolism	hsa00230	8833 51292 3251 27115 8382 1633 10621
